# Is Autosomal Dominant Polycystic Kidney Disease Becoming a Pediatric Disorder?

**DOI:** 10.3389/fped.2017.00272

**Published:** 2017-12-20

**Authors:** Stéphanie De Rechter, Luc Breysem, Djalila Mekahli

**Affiliations:** ^1^PKD Lab, Department of Development and Regeneration, KU Leuven, Leuven, Belgium; ^2^Department of Pediatric Nephrology, University Hospitals Leuven, Leuven, Belgium; ^3^Department of Radiology, University Hospitals Leuven, Leuven, Belgium

**Keywords:** autosomal dominant polycystic kidney disease, children, testing, prevention, treatment

## Abstract

Autosomal dominant polycystic kidney disease (ADPKD) affects 1 in 400 to 1,000 live births, making it the most common monogenic cause of renal failure. Although no definite cure is available yet, it is important to affect disease progression by influencing modifiable factors such as hypertension and proteinuria. Besides this symptomatic management, the only drug currently recommended in Europe for selected adult patients with rapid disease progression, is the vasopressin receptor antagonist tolvaptan. However, the question remains whether these preventive interventions should be initiated before extensive renal damage has occurred. As renal cyst formation and expansion begins early in life, frequently *in utero*, ADPKD should no longer be considered an adult-onset disease. Moreover, the presence of hypertension and proteinuria in affected children has been reported to correlate well with disease severity. Until now, it is controversial whether children at-risk for ADPKD should be tested for the presence of the disease, and if so, how this should be done. Herein, we review the spectrum of pediatric ADPKD and discuss the pro and contra of testing at-risk children and the challenges and unmet needs in pediatric ADPKD care.

## Autosomal Dominant Polycystic Kidney Disease (ADPKD) and Its Spectrum in Pediatric Patients

Autosomal dominant polycystic kidney disease affects 1 in 400 to 1,000 live births, making it the most common monogenic cause of renal failure and representing a major socio-economic medical problem in the world (1). The disorder arises as a consequence of *PKD1* or *PKD2* gene mutations, encoding polycystin-1 (PC1) and -2 (PC2), and accounting for 85 and 15% of patients with a *PKD* gene mutation, respectively. Recently, a third gene has been identified to cause ADPKD when mutated, namely *GANAB* (Glucosidase, Alpha, Neutral AB form), encoding the glucosidase IIa subunit (2). Although most cases are familial, in 10–25% of patients, a positive family history is absent, posing a diagnostic challenge. These cases are explained by *de novo* disease in up to 10–15% (3), missing parental medical records, germline or somatic mosaicism, or mild disease from hypomorphic *PKD1* and *PKD2* mutations (4).

Autosomal dominant polycystic kidney disease is characterized by a progressive growth of cysts in all nephron segments, ultimately leading to end-stage renal disease (ESRD) in 50% of patients in the sixth decade. ADPKD patients with a *PKD2* mutation have a milder phenotype and reach ESRD approximately 20 years later than *PKD1* patients. Within the *PKD1* population, patients with a truncating mutation are more severely affected than those with a non-truncating. Therefore, *PKD* gene mutation analysis is one of the elements forming the PROPKD scoring system, an algorithm to predict renal survival in adult ADPKD patients (5). Moreover, ADPKD as a bona fide ciliopathy is a systemic disorder, comprising extrarenal symptoms such as cyst formation in other organs, mostly in the liver, hypertension, intracranial arterial aneurysms, cardiac valvular defects, inguinal and abdominal herniation, etc. (6). Recently, there is also evidence of bone and mineral metabolism anomalies, from both studies in different animal ADPKD models (7–9) as from clinical studies in adult and pediatric patients (7, 10–14).

As the majority of patients remain *a-* or *pauci-*symptomatic until adulthood, ADPKD is still often considered as a late-onset disease (15). Historically, ADPKD was termed “adult” polycystic kidney disease due to this classic presentation (16). However, comorbidities might be underdiagnosed in childhood if not actively looked for by caregivers as not all cause complaints. Moreover, the glomerular filtration rate (GFR) masks the underlying renal damage for several decades, due to hyperfiltration and hypertrophy of unaffected residual nephrons (17). The parameter proven to be a strong and independent predictor for ESRD development in ADPDK patients was the factual anatomic damage, indirectly assessed as the height-adjusted total kidney volume (TKV) on magnetic resonance imaging (MRI) (18, 19). Although evidence is growing that renal injury starts with the formation of the first renal cysts *in utero* (20), a consensus for the management of children at-risk for or diagnosed with ADPKD is lacking. Indeed, of all ADPKD patients, 2–5% present in childhood with a broad phenotypic spectrum, ranging from severe neonatal presentations mimicking autosomal recessive polycystic kidney disease (21) to the incidental sonographic finding of renal cysts (22). Here, we aimed to summarize the most important manifestations of ADPKD in childhood.

### Renal Manifestations in Childhood

Possible presenting symptoms of renal disease in children with ADPKD are frequency, nocturia and/or, hematuria, urinary tract infection(s) and back, flank or abdominal pain (23). Often, the earliest biological renal manifestation of childhood ADPKD is a decreased urinary concentrating ability, resulting in polyuria and polydipsia (15). 58% of children (*N* = 53) showed a decreased renal concentrating capacity when performing a standardized renal concentrating capacity test after nasal drop application of desmopressin (24). In several study cohorts, children were included having a decreased estimated GFR (eGFR): 12 (25) to 39% (26) of the ADPKD children had eGFR levels < 90 mL/min/1.73 m^2^, and ESRD was seldom observed. Indeed, cases of ESRD in childhood due to ADPKD are rare and in these cases, coexisting reasons for renal function decline should be ruled out. Other reasons might be the coinheritance of a hypomorphic *PKD1* allele in trans with an inactivating *PKD1* allele or the inheritance of two incompletely penetrant *PKD1* alleles—most likely leading to very early onset (VEO) ADPKD (27, 28). Children with ADPKD display glomerular hyperfiltration in 18 (29) to 21% (25). Importantly, the occurrence of hyperfiltration was associated with a significantly faster renal function impairment and a higher renal growth rate over time (29). Also, proteinuria and microalbuminuria are seen in ADPKD, and more frequently in children and adolescents than in adults. In different pediatric cohorts, the prevalence of micro-albuminuria and proteinuria ranges from 30 to 48% and 10 to 23%, respectively (25, 26, 30). From studies performed in both pediatric (30) and adult (31) patient cohorts, it was shown that patients with established proteinuria have a more aggressive course of renal disease. Indeed, adult proteinuric patients had higher mean arterial pressures, larger renal volumes and lower creatinine clearances compared to the non-proteinuric subjects (31). Similarly, proteinuric children had a more severe renal disease -defined as having more than 10 renal cysts—then those without proteinuria. However, unlike in adults, no correlation between proteinuria and hypertension, one of the most prevalent extrarenal manifestations, was seen in children (30). Last, hematuria, both possible after an antecedent of abdominal trauma, for instance in patients playing contact sports, as spontaneously (32), and nephrolithiasis are rather uncommon in children (15). A prevalence of the latter is lacking, as information comes from case reports (33). Moreover, nephrolithiasis is seen in up to 28% of ADPKD patients, which is 5–10 times the rate in the general population, due to both anatomic and metabolic abnormalities; but none in the patients below 20 years of age (34). Different from the general population is that nephrolithiasis in ADPKD patients should only be made after ruling out a superimposed urinary tract infection, cyst infection or hemorrhage and that screening for underlying urinary metabolic abnormalities should be performed from the first presentation (35).

The anatomic renal damage can often already be seen from a young age as renal cysts and/or uni- or bilateral nephromegaly. In a cohort of 47 ADPKD children, all had accelerated renal growth relative to the normal population, and half had nephromegaly (26). Affected individuals who do not yet have cysts detectable by ultrasound (US) were shown to have larger age-adjusted kidneys, suggesting ongoing microscopic cyst development and growth (36). Importantly, mathematical models computing volume changes of solitary and multiple cysts in function of time show that cysts formed early in life, are the main contributors to the final total cyst volume (37).

Another major symptom of childhood ADPKD, explained by anomalies in both the renal and cardiovascular system, namely hypertension, will be discussed in Section “Extrarenal Manifestations in Childhood: Cardiovascular Symptoms.”

### Extrarenal Manifestations in Childhood: Cardiovascular Symptoms

Cardiovascular manifestations are the most prominent extrarenal manifestation in ADPKD, also in childhood, of which hypertension occurs in a majority of patients, prior to renal function decline. The reported incidence in childhood varies widely in different cohorts, namely from 5 to 44% (15): 6% (25), 15% (26), 21% (30), 33% (38), and 37% (29). The most reliable assessment probably is a recently published meta-analysis covering 14 studies, estimating 20% of ADPKD children having hypertension (39).

The pathogenesis of hypertension in ADPKD includes a complex and multifactorial process, involving the renin–angiotensin–aldosterone system (RAAS), the sympathetic nervous system, sodium retention, and endothelial dysfunction. First, the presence of growing renal cysts was shown to attenuate intrarenal blood vessels, suggesting the presence of local renal ischemia and hypoxia. This in turn suggests stimulation of the RAAS in ADPKD. Both increased circulating (40) as intrarenal (41) RAAS activity was demonstrated in patients with ADPKD. Second, it is known that RAAS activation stimulates the sympathetic nervous system and *vice versa*. Indeed, adult hypertensive ADPKD patients have significantly higher plasma catecholamine concentrations compared to patients with essential hypertension (42). Third, sodium retention plays a role. Interestingly, adult patients with normotension were shown to exhibit an increased total body sodium as compared with their unaffected siblings, suggesting an early role of RAAS (43). Last, expression of Pkd1 and Pkd2 was shown in the major vessels and in the cilia of endothelial and vascular smooth muscle cells. Decreased or absent PC1 or -2 levels are associated with abnormal vascular structure and function, *via* reduced nitric oxide (NO) levels due to diminished Ca^2+^-dependent endothelial NO syntheses activity, resulting in an altered endothelial response to shear stress with attenuation in vascular relaxation. Moreover, decreased NO production leads to the activation of the RAAS, next to its triggering by cyst enlargement and intra-renal ischemia (44). However, it is still unclear which factor hypertension in ADPKD is mainly due to. The early onset of hypertension in ADPKD, probably still underdetected and therefore left untreated, is associated with left ventricular hypertrophy (LVH) in half of the patients in their 40s (45). Importantly, cardiovascular complications appear to be the leading cause of death in patients with ADPKD. Therefore, LVH should be prevented in patients with ADPKD, as it is known to be associated with coronary heart disease, cardiac failure, arrhythmias, and sudden death (46).

A correlation between TKV and cyst volume, and hypertension was observed in a longitudinal MRI study in ADPKD children: the hypertensive subgroup had a larger increase in both cyst volume and number than the normotensive (47). Also, the prevalence of hypertension was found to be significantly higher in children with a decreased urinary concentrating capacity compared to those with a normal concentrating capacity. Significant negative correlations were shown between urinary concentrating capacity, blood pressure and the number of renal cysts (24).

Vascular dysfunction begins early in the course of ADPKD. In normotensive patients with preserved renal function, the endothelium-dependent dilatation and arterial stiffness were evaluated and compared to age and sex matched healthy controls, by measuring the brachial artery flow-mediated dilation (FMD_BA_) and the carotid-femoral pulse wave velocity (CFPWV), respectively. FMD_BA_ was lower in 25% of patients, indicating an impaired endothelium-dependent dilatation, while CFPWV was higher in 14% of patients, designating increased arterial stiffness. Both are well-known independent predictors of future cardiovascular comorbidity and mortality (48).

Another possible cardiovascular feature in ADPKD, is mitral valve prolapse, occurring in 12% of children (15). Next, the association of ADPKD with intracranial aneurysm formation is well known (49). Almost all cases of ruptured intracranial aneurysms occur in adult patients with a median age at rupture of 40 years. In a study screening ADPKD patients, aged 7–87 years, the prevalence of intracranial aneurysm was 12.4%, reaching a peak in the 60–69 years age group; and higher in patients with a positive family history of hemorrhagic stroke or intracranial aneurysm. Only 1 patient had an aneurysm before the age of 29 years, but the authors do not state the exact age of this patient (49). However, case reports describe rupture at very young ages, even neonatal (50, 51). Although rare, this complication might be lethal in young patients.

### Extrarenal Manifestations in Childhood: Others

Recently, evidence has emerged showing bone involvement in ADPKD (7–14). Indeed, PC1 is highly expressed in both osteoblasts and osteocytes and a homozygous *PKD1* null mutation in mice was shown to cause polyhydramnios, hydrops fetalis, spina bifida, and osteochondrodysplasia (52, 53). Moreover, the PC1 C terminal tail regulates TAZ expression, an enhancer of RUNX2-mediated osteoblast differentiation and suppressor of PPARγ-stimulated adipocyte differentiation. A causal relationship between mutant PC1, decreased Runx2 levels, and impaired osteoblast differentiation leading to a defect skeletogenesis was described (9, 54).

It has been demonstrated that fibroblast growth factor 23 (FGF23), a phosphaturic hormone secreted by osteocytes, was studied in different ADPKD rodent models. FGF23 levels were raised, but a peripheral biologic resistance to FGF23 was hypothesized to account for the absence of a renal phosphate leak or hypophosphatemia. Importantly, FGF23 was detected in cells lining renal cysts, from a rodent model (7). In ADPKD adults in chronic kidney disease (CKD) stages 1–2, FGF23 levels were highly increased compared to both healthy controls and CKD matched controls. However, the majority of the ADPKD population displayed FGF23 resistance, given the absence of a phosphaturic phenotype, confirming the observations from the animal models. This was hypothesized to be due to the deficiency of the FGF coreceptor, namely serum Klotho (sKlotho) (13). FGF23 has also been studied in ADPKD children with preserved renal function. Compared to healthy controls, patients had significantly lower serum phosphate SDS. In a quarter of the patient population, this was accompanied by low TmP/GFR levels for age, based on the reference values (55). Although FGF23 levels were similar in both groups, it is inappropriately normal for the ADPKD cohort, given the hypophosphatemia in the latter. Also sKlotho levels were similar between both groups. Apart from the phenotypic differences in adulthood vs. childhood concerning FGF23 and phosphate metabolism, these anomalies seem to be ADPDK specific, as markedly FGF23 elevation is only found in non-ADPKD patients with advanced CKD stage, where it is thought to counteract phosphate retention and is classically accompanied by an increased parathyroid hormone and decreased 1,25-dihydroxyvitamin D levels (14). Moreover, given the observations in the pediatric cohort, it is an early disease phenomenon.

In the same pediatric ADPKD cohort, significantly lower values of bone alkaline phosphatase were seen compared to healthy controls (10). Based on these data and the findings from a bone biopsy study in which a low bone turnover was observed in young adult ADPKD patients with preserved kidney function (56), bone formation suppression is hypothesized in ADPKD.

Also sclerostin, known to inhibit the osteo-anabolic Wnt/β-catenin pathway, is an emerging player in the mineral metabolism phenotype of ADPKD. Higher osseous sclerostin expression has been reported in *jck* mouse model (57) and in a small cohort of adult ADPKD patients in ESRD (11). However, it could not be confirmed in a pediatric ADPKD cohort (10). This discrepancy remains to be elucidated, next to the clinical relevance of sclerostin overactivity in ADPKD, if confirmed in future studies.

In general, one of the main extrarenal manifestations of ADPKD is polycystic liver disease (PLD). In contrast to renal cyst formation, liver cyst formation does not affect liver function, although it can cause symptoms related to its mass effect when significant liver enlargement has occurred, or due to cyst bleeding or infection (58). In children, liver cyst formation is very rare (59). PLD is the result of ductal plate—the primitive biliary structure—malformations. Normally, ductal plate remodeling takes place from 12 weeks gestational age, up to term, but with a slowing down between the 20th and the 32nd week of gestation (60). Ductal plate malformation leads to a partial to complete arrest of ductal plate remodeling, and therefore a persistent excess of embryonic biliary structures (61). Typically, these structures are present from a very early disease stage, but remain asymptomatic until cyst growth initiates in adulthood (62).

## Diagnosing ADPKD in Children

The diagnosis of ADPKD in children can be made either unintentionally or actively, *via* screening. First, children at-risk for ADPKD might present with symptoms such as urinary tract infection(s), hematuria, enuresis etcetera, leading to their diagnosis when further investigated. Children might be diagnosed unintentionally as well, starting from the routinely performed prenatal US during pregnancy. Prenatally, the typical—although not specific for ADPKD—sonographic characteristics are moderately enlarged hyperechogenic kidneys with an increased corticomedullary differentiation (CMD). Other prenatal sonographic features such as an absent or decreased CMD and cortical cysts are less frequently seen (21). Although a prenatal presentation of ADPKD is rare, this form is recurrent within families, suggesting a common genetic modifying background with low levels of PC1 function (63) or the association with other modifying genes such as *HNF1B* or *PKHD1* (64). In other cases, children might be diagnosed unintentionally postnatally, after the coincidental detection of renal cysts on an abdominal US performed for other reasons, most frequently abdominal pain. Second, active presymptomatic testing might be requested by the parents and/or the adolescents themselves. However, the diagnosis of ADPKD, especially its timing, represents controversies that we will discuss later in this review.

We first aim to demonstrate the challenges and limitations in the methods for the diagnosis of ADPKD in children and the lack of definite criteria for individuals under the age of 15 years. Indeed, presymptomatic testing in children at-risk for ADPKD, due to their positive familial history, can be executed by clinical evaluation and/or imaging. However, genetic evaluation is needed to resolve some cases. Most diagnoses of ADPKD are established on a positive family history combined with imaging [sonographic (65, 66) or MRI (67)] findings. However, for individuals under the age of 15 years, there are no validated diagnostic imaging criteria available (36, 68). As simple renal cysts occur rarely in childhood (69), a child with a positive family history for ADPKD, having a single cyst, preferably > 1 cm in diameter, is generally assumed to have the diagnosis of ADPKD, although this was never fully evaluated and validated. Moreover, based on renal US findings, one can only exclude disease presence in at-risk individuals aged 30–40 years or older (65–67). Based on MRI, this will probably be possible at an earlier age, but a cutoff age for a definite exclusion is not set yet. In case of a negative US and the patient and their family are willing to have a more definitive evaluation, gene sequencing of the *PKD* genes can be performed. However, this is not yet routinely used in clinical practice yet due to its high cost and challenging analysis. These are explained by the presence of six *PKD1* pseudogenes and the remarkable allelic heterogeneity. While the diagnostic accuracy of *PKD1* and *PKD2* gene mutation testing was found to be lower than the accuracy of US examination in adults above the age of 30 years, the relative accuracy of genetic vs. sonographic testing was comparable for children younger than 15 years in *PKD1* and superior in *PKD2* individuals (65). Also, in case the familial mutation has been identified, it is more cost effective to sequence the exon with the known mutation in at-risk individuals of this specific family. However, in that case, there is still a chance of a non-familial *de novo* mutation, not completely excluding the disease. Also, in case of a non *PKD1* non *PKD2* family, in which there is a clear ADPKD family history, but the *PKD* gene mutational analysis remains negative, exclusion of the disease in at-risk individuals will be possible only by means of a negative renal US at the age of 30–40 years or older (70).

Importantly, children who were diagnosed *in utero* or within their first 18 months of life, the so-called VEO group, represent a particularly high-risk group of ADPKD patients and should be managed accordingly. Indeed, this group was shown to have more hypertension and progression of eGFR < 90 mL/min/1.73 m^2^ by the last follow-up at the median age of 16 years; although they had similar rates of glomerular hyperfiltration compared to a non-VEO ADPKD control group (27).

## The Controversy of Testing At-Risk Children for the Presence of ADPKD

Currently, apart from the Kidney Disease: Improving Global Outcomes (KDIGO) Controversies Conference Report (71) and the European ADPKD Forum (EAF) Report (72), no guidelines are available for the clinical care of ADPKD families.

In the KDIGO consensus, it is recommended to have at-risk children screened for hypertension, starting at the age of 5 years, and repeated every 3 years in case of normotension. Presymptomatic screening of ADPKD by means of US or genetic analysis is not currently recommended for at-risk children, while it is accepted for at-risk adults, in whom the potential benefits of presymptomatic diagnosis usually outweigh the risks. However, on the other hand, this consensus suggests the following three options for at-risk children to be discussed thoroughly with their parents, so the parents can decide which approach will be taken. First, to screen the children and to disclose the results to the whole family; second to screen the children and disclose the results only to their parents, or last, not to screen the children. Moreover, according to the consensus, molecular genetic testing should be considered in cases of atypical renal imaging, discordant disease within a family; very mild PKD; in a patient without a family history for PKD; early and severe forms of PKD or PKD associated with syndromic features; and reproductive counseling (71).

In the EAF report, it is not discussed whether at risk children should be tested, although it is stated that genetic testing is vital for diagnosing ADPKD in children and that genetic counseling including informing the patients on the possibility of preimplantation genetic diagnosis for ADPKD should be offered at initial diagnosis (72).

Taken together, it is still a matter of controversy whether asymptomatic individuals at-risk for ADPKD, especially minors, should be tested for the presence of the disease or not (73). However, they and their family should be informed about the possibility of testing, and reasons why (not) to test. An important contra to testing, is the current absence of a cure (6). Although over the last decade, several drug targets based on the cellular mechanisms were tested in *in vitro* and *in vivo* models to slow down cystogenesis, only one drug demonstrated a moderate effect on disease progression in adult ADPKD patients, namely the vasopressin V2 receptor antagonist tolvaptan (74), as also concluded by a recent Cochrane review on interventional studies in adult patients (75). Despite its adverse effects of polydipsia and polyuria, it is currently the only drug available in Europe, indicated in selected adult patients with CKD stages 1–3, having evidence of rapidly progressing disease (76). Important to take into account are the methodological issues, limiting the interpretation of results of the published trials up till now. Though these are often encouraging, they are frequently restricted because of inadequate power, short study duration, patients’ heterogeneity (large differences in TKV and/or underlying mutation class for instance), doses with inadequate pharmacological effects and uncertain target organ concentration (77). Moreover, the question remains whether it would be more efficacious to start treatment before extensive renal damage has occurred. A second contra is the potential induction of psychological stress in an at-risk individual and his/her family, possibly causing feelings of (survival) guilt; although not only the certainty of having a diagnosis might be experienced as unbearable, but also the uncertainty when not testing (78, 79). A recently published mono-center survey performed in adults diagnosed with ADPKD, prior to the start of renal replacement therapy, showed that 22% had a clinically significant depression and 62% felt guilty about passing the disease on to their children. The latter represented one of the major psychological challenges confronting patients with ADPKD (80). A third argument against presymptomatic testing is the negative financial consequences a diagnosis with ADPKD might cause, such as difficulties obtaining insurances (81).

On the other hand however, presymptomatic testing not only has a prognostic value, by means of TKV measurement and *PKD* genotyping; it may also give caregivers the opportunity to target modifiable risk factors for disease progression from an early disease stage (82), thereby improving long-term renal outcome (71). This is supported by the observation of similar outcomes in a study comparing the presence of nephromegaly, hypertension, microalbuminuria and decreased eGFR between a cohort in which diagnosis was based on postnatal US screening vs. after presenting symptoms (26). This emphasizes the importance of regular screening for hypertension and proteinuria in at-risk children, but could also be seen as supporting argument for presymptomatic testing. Indeed, the significance of early treatment of disease symptoms should not be neglected. In children, this was demonstrated by slower cyst growth in ADPKD children with normotension compared to those with hypertension (47). Moreover, effective blood pressure control from childhood on may ameliorate the cardiovascular outcomes in these patients, known to be at high risk for early cardiovascular events (83, 84). Although glomerular hyperfiltration was shown to correlate with a more severe renal phenotype (29), there is no evidence (yet) for regular screening and/or treatment of it. Also, for some identified disease progression markers in adults, it remains to be determined to what extent their modification may influence the clinical course of ADPKD, such as for higher urinary sodium excretion and lower serum HDL cholesterol (85). A benefit accruing to all tested individuals, is their empowerment, namely the process through which people gain greater control over decisions and actions affecting their health and life, an important aspect is informed reproductive decision making (79).

A survey performed among European pediatric and adult nephrologist and geneticists before the KDIGO publication and the EMA approval of tolvaptan, demonstrated that their clinical practice and beliefs are very heterogeneous regarding testing of asymptomatic offspring and other ethical issues in the management of ADPKD families (86). In this study, agreement between respondents was observed regarding clinical testing of at-risk minors, although pediatric nephrologists supported this significantly stronger compared to geneticists. Also, the three groups of caregivers showed a moderate disagreement on the performance of genetic testing for ADPKD in at-risk minors. However, the differences observed in their counseling attitude were only attributable to their professional discipline. Therefore, ADPKD patients and their family tend to receive conflicting and/or incomplete information from their caregivers. As suggested in the article, a reevaluation in a few years from now, to evaluate how the KDIGO consensus statement and the availability of tolvaptan and probably other upcoming treatment options influence both professionals and patients beliefs on these issues, would be interesting to perform (86).

## Childhood as a Critical Therapeutic Window for (Future) Therapies

As suggested earlier, we should ask ourselves whether the best chance for preserving long term renal function in ADPKD is to take an early start. Starting prevention before extensive renal damage has occurred, thus at a young(er) age, might indeed improve the long term renal survival. Moreover, as suggested by Grantham et al., based on the assumption that decline in GFR is linked to the progressive increase in TKV, early treatment has the potential to delay renal dysfunction progression by more than a decade. When therapy is started at 18 years of age, reduction in the rate of TKV growth is predicted to shift from 5 to 2.5%, or 1.25% per year—a change that yields a vastly different effect on GFR decline than that induced by starting therapy later in life (87).

However, before heading toward cystogenesis inhibiting treatment options in children, disease manifestations requiring symptomatic treatment should be identified and managed adequately (16).

In both children and adults with ADPKD, hypertension is a predictor of worse renal outcome. Moreover, it is associated with increased cardiovascular morbidity and mortality (15).

In adults with ADPKD, LVH, a major cardiovascular risk factor, was significantly further decreased by rigorous (<120/80 mmHg) than standard blood pressure control (<140/90 mmHg), although this was not associated with a statistically significant difference in renal function between the two treatment arms (88). Moreover, from the HALT/PKD study, it was shown in an early ADPKD cohort that as compared with standard blood pressure control (120/70 to 130/80 mmHg), rigorous blood-pressure control (95/60 to 110/75 mmHg) was associated with a slower increase in TKV, no overall change in the eGFR, a greater decline in left ventricular mass index, and a greater reduction in albuminuria levels (89). These data may be particularly applicable to children as the study cohort represented early stages of ADPKD. However, a study using angiotensin converting enzyme inhibitors (ACEi) in children and young adolescents with ADPKD, over a period of 5 years, failed to demonstrate a significant effect on renal growth in the total cohort. Importantly, ACEi treatment was associated with stable renal function and left ventricular mass index in the (borderline) hypertensive children, who are at particular risk for increases in renal volume and left ventricular mass index and decreased renal function as compared with the other study groups (90).

KDIGO states that treatment of hypertension in pediatric ADPKD patients should follow prevailing pediatric guidelines; meaning a goal blood pressure below the 90th percentile for age, sex, and height; with the only exception that RAAS blockade is preferred as first-line treatment (71). However, given these findings, it was recommended recently to use an ACEi with a target blood pressure of <110/75 in young adults older than 18 years; and in adolescents and children with borderline hypertension (75–95th percentile) or hypertension (≥95th percentile), to use an ACEi to achieve a goal blood pressure ≤50th percentile. Angiotensin receptor blockers (ARBs) can be used in patients who do not tolerate ACEi (15). Combination of both RAAS blockers should be avoided as no benefit was demonstrated by adding an ARB (telmisartin) to an ACEi (lisinopril) on ADPKD progression in adult patients with early or moderately advanced kidney disease (89). Moreover, girls of childbearing potential and their families should be informed on fetal toxicity of ACEi and ARBs (15).

Next, in a 3–year, phase III clinical trial of pravastatin on TKV and left ventricular mass index, pravastatin was effective in slowing the progression of structural kidney disease in older children and young adults with ADPKD. There was no beneficial effect on the left ventricular mass index, nor on their urinary micro albumin excretion (91). However, a recently published *post hoc* analysis of the HALT PKD trials, comparing the outcomes of participants who never used statins with those who used statin for at least 3 years, failed to demonstrate a benefit of statin therapy in both subjects with a preserved renal function (Study A) as a reduced renal function (Study B) (92). Conclusions remain preliminary, as the first study consisted of a relatively small cohort, and the *post hoc* analysis of the HALT PKD is limited given the small number of statin users in Study A, different statin drugs and doses used, non-randomized allocation and advanced disease stage in Study B. Also, as with ACEi, potential risks to the fetus with statin exposure during pregnancy should be reviewed prior to treatment to females of appropriate pubertal development.

As mentioned before, currently, the only drug available for selected (<50 years, CKD stages 1-3a, rapidly progressing disease) adult patients in Europe (76) is the vasopressin V2 receptor antagonist tolvaptan. Tested in the TEMPO clinical trials, including adult patients with a relatively advanced disease stage (TKV > 750 mL), tolvaptan use was demonstrated to induce both a slower annual increase in TKV and a slower annual decline in kidney function in treated patients vs. controls (74). Despite its possible adverse effects—polydipsia, polyuria and significant liver enzyme elevation—tolvaptan is currently studied in a phase III double-blind placebo controlled clinical trial in a pediatric ADPKD cohort within Europe (NCT02442674) (93). Apart from this, there is one case report, describing the off-label use of tolvaptan in a severely affected neonate with ADPKD (94). Other coming exciting therapies, such as the AMPK activator metformin, are currently tested in adult patients, and will be tested in pediatric ADPKD depending on the results in adults (95). A general remark on (future) studies including children with ADPKD is that the subjects often are performed in tertiary and specialist centers. This might reflect highly selected populations, likely to contain a higher proportion of symptomatic children and “fast-progressors,” influencing the results.

## Challenges and Unmet Needs in Pediatric ADPKD Care

In the care of pediatric ADPKD and their families, there is a clear need of a more standardized management. Few initiatives have been taken so far, namely the KDIGO consensus and EAF report, however, conflicting recommendations still need standardization and completion such as testing of at-risk minors and the target blood pressure when treating hypertension (50th vs. 90th percentile) as discussed earlier. Moreover, there are no details provided on how blood pressure should be monitored, namely *via* an office blood pressure measurement or a 24-h ambulatory blood pressure monitoring. There is evidence that the latter should be performed routinely from a large pediatric cohort, in which 51.9% of patients lacked a physiological nocturnal blood pressure dipping and 18.2% had isolated nocturnal hypertension (Massella L et al. High prevalence of hypertension in a European cohort of children with ADPKD: results of the ADPKiDs study. IPNA; 2016; Iguacu, Brazil) (96).

Others recommendations are absent, such as on screening for intracranial aneurysms, both in adults and in children, a topic in which many areas of uncertainty remain, as observed in a recent survey among European French speaking nephrologists (97). However, there is recent evidence that systematic screening in all ADPKD patients, regardless of the family history for intracranial aneurysms, is cost-effective (98). Moreover, despite the recommendations, it will take time to implement these. Till now, many at risk children with ADPKD are not under regular follow-up and remain undiagnosed (39). Thus, leaving the important issue who should be responsible for informing them in adulthood: only the family, both the family and the caregivers or only the caregivers? From the caregivers’ point of view, this is a shared responsibility for professionals with the family (86), although it would be interesting to know the opinion of ADPKD patients on this.

Next, there is a need of diagnostic imaging criteria under the age of 15 years apart from the currently used eminence-based guidelines. The most important is the need for clear prognostic criteria from childhood, as a validated method to identify slow vs. rapid progressors does not exist in the pediatric population, except in case of VEO ADPKD (27). However, this is necessary for the stratification of young patients in clinical trials, and possibly as new and better outcome measures. Indeed, TKV as a prognostic marker is well-assessed and accepted, but only in studies starting to include patients from the age of 15 years (87). Moreover, TKV measurements *via* MRI were shown to be more accurate and reproducible compared to US in adults (99), however, unfeasible in many young children due to the need of sedation or general anesthesia. Therefore, new modalities should be tested for TKV measurement in ADPKD children. One promising option is a three dimensional US technique, which was shown a valuable alternative to MRI to measure, although underestimating MRI TKV (96, 100).

Untill now, most studies published were performed on small pediatric cohorts, while we need to build large cohorts before we will be able to define evidence-based recommendation for children with ADPKD. Therefore, we recently have launched our initiative of the “ADPedKD” international registry to collect retrospective and prospective longitudinal clinical, imaging, and genetic data of children until the age of 19 years.

## Conclusion

In conclusion, children and young adults with ADPKD may represent a critical therapeutic window to reduce future renal and cardiovascular risk. However, we are still far from standardized care for this population. Moreover, the ultimate goal should be to empower patients to make their own informed decisions.

According to the KDIGO consensus, the advised follow-up of at-risk children should encompass regular monitoring for disease manifestations such as hypertension and proteinuria, so treatment of these disease modifying factors is initiated as early as possible.

## Author Contributions

The authors confirm being the sole contributors of this work and approved it for publication.

## Conflict of Interest Statement

The authors declare that the research was conducted in the absence of any commercial or financial relationships that could be construed as a potential conflict of interest.

## Abbreviations

ACEi, angiotensin converting enzyme inhibitor; ADPKD, autosomal dominant polycystic kidney disease; ARB, angiotensin receptor blocker; CKD, chronic kidney disease; (e)GFR, (estimated) glomerular filtration rate; LVH, left ventricular hypertrophy; MRI, magnetic resonance imaging; RAAS, renin–angiotensin–aldosterone system; TKV, total kidney volume; VEO, very early onset; US, ultrasound.
